
JOURNAL INFORMATION
==============================
NLM Title Abbreviation: Inter Econ
Journal ID: Inter Econ
NLM Journal ID: 101086399

Inter Economics

ISSN: 0020-5346

ARTICLE INFORMATION
==============================
PMCID: PMC7385200
PMID: 32834102
DOI: 10.1007/s10272-020-0912-2
Article ID: 912
Article version: 1
Subjects: Letter from America

Early Care and Education: Necessary Infrastructure for Economic Recovery

Appelbaum Eileen appelbaum@cepr.net

grid.417588.7 0000 0004 0398 7584 Center for Economic and Policy Research, 1611 Connecticut Avenue, 20009 Washington, DC, USA
Electronic publication date: 2020 Jul 28
Print publication date: 2020
Volume: 55
Issue: 4
First page: 271
Last page: 272
Copyright: © The Author(s) 2020
License: Open Access This article is licensed under a Creative Commons Attribution 4.0 International License, which permits use, sharing, adaptation, distribution and reproduction in any medium or format, as long as you give appropriate credit to the original author(s) and the source, provide a link to the Creative Commons licence, and indicate if changes were made.
The images or other third party material in this article are included in the article’s Creative Commons licence, unless indicated otherwise in a credit line to the material. If material is not included in the article’s Creative Commons licence and your intended use is not permitted by statutory regulation or exceeds the permitted use, you will need to obtain permission directly from the copyright holder.
To view a copy of this licence, visit http://creativecommons.org/licenses/by/4.0/.
License URL: https://creativecommons.org/licenses/by/4.0/

issue-copyright-statement: © The Author(s) 2020

==============================

The coronavirus pandemic has brought into bold relief the essential nature of care work for families and the economy. As feminist economists have long argued, care jobs that in the U.S. are done by predominantly female and disproportionately Black, Latinx and immigrant workers are the backbone that sustains our communities and undergirds our economic prowess. COVID-19 has revealed wide racial, ethnic and class disparities in access to health and other care services that can no longer be ignored. Americans cannot deny the low pay and unsafe working conditions facing workers we now salute as heroes. In the U.S., the reckoning with our history demanded by the Black Lives Matter movement means we cannot pretend we do not see the origins of these inequities in slavery. Enslaved Black women were made to care for their owners' homes and family members, including their white children.

After slavery ended, Black women had few employment options and continued to do domestic and care work. The Depression-era law that established minimum wage and other worker protections excluded occupations that employed large numbers of Black workers, including those doing child care. The jobs were viewed as requiring no special expertise and were low paid. Later, when the care of young children moved from the home to more formal settings, and as Black women were joined by immigrant care workers, slavery-era attitudes toward this work and the women who perform it persisted.

Child care centers and family child care homes are among the organizations that have suffered the greatest revenue losses as COVID-19 shut down businesses and halted much economic activity. The pandemic led to the widespread closings of facilities that care for young children. A survey from the National Association for the Education of Young Children conducted during the first week of April found that nearly half of these facilities were completely closed. The Bureau of Labor Statistics reported a decline in employment in child daycare services from 1.02 million workers in March 2019 to 688,000 in April 2020 before recovering somewhat to 765,000 in June, a 25% decline from its pre-pandemic level. Early evidence suggests that the stay-at-home orders mainly affected employment in for-profit and nonprofit facilities where care is paid for by parents, with subsidies for very low-income households. Government-sponsored programs were not affected. The situation may be even more dire. There are 900,000 grandparents, family members and nannies who provide informal care that may not be able to continue due to COVID-19.

A sharp decline in child care slots in a system that was already inadequate threatens the return of millions of workers, mainly women. In December 2019, women made up more than half the workforce; now, for the first time in a recession, women have lost jobs at a more rapid rate than men. Their return to employment is essential if the U.S. economy is to recover from the pandemic. Public financing to restore the pre-pandemic level of childcare services is the first step in assuring that all workers – including the nearly two-thirds of mothers with a child at home under the age of six who were in the labor force in 2019 – can return to work.

Economists Rebecca Ullrich and Aaron Sojourner have estimated the costs of maintaining existing formal child care infrastructure during the pandemic. Funding facilities that closed would cost $3.9 billion per month. The monthly cost to support child care providers who remained open is $6.3 billion, a total of nearly $10 billion monthly or $50 billion for five months. The $2 trillion government bailout in March provided $500 billion for industry, including $46 billion earmarked for airlines. The child care industry, which requested $50 billion, was allocated just $3.5 billion. On July 16, Senate Democrats introduced a bill to reprogram $200 billion of still unspent bailout funds to meet demands for economic justice, with $50 billion to stabilize child care providers.

Investment in infrastructure serves as both economic stimulus in the short run and a boost to productivity growth in the long run. Infrastructure investment is identified broadly with investment in physical structures – roads, bridges, airports, waste management facilities and so on. But investment in social infrastructure shares many of the characteristics that make physical infrastructure valuable. It is job creating, job enabling and provides a solid foundation for the subsequent success of children and their future economic contributions.

A recent study by Jerome De Henau and Susan Himmelweit examined the *job creating* potential of investing in care infrastructure. The researchers found that investments in the care of young children and in long-term care of the elderly created far more jobs than investing in construction. This is due to the fact that care industries are much more labor intensive than construction, which makes far greater use of capital goods to produce output. Of course, care jobs are more likely to be part-time than construction jobs and to pay much lower wages. The researchers accounted for that by considering a situation in which the care jobs were good jobs, paying much higher wages than is currently the case. In Sweden, wages of child care and long-term care workers are 87% of construction worker wages; in the U.S., reflecting the historical origins of this work, they are just 44%. By comparison, in Germany they are 67% of construction worker wages, in Italy they are 70% and in Spain, 72%. Researchers carried out their analysis assuming that wages of care workers in the U.S. were 87% of U.S. construction worker wages. They found that a similar amount of spending in the construction and care industries would lead to an increase in employment that was 40% higher in care than in construction. For every 100 jobs created by such spending on construction, 140 jobs were created by spending on care. Most of the increased employment occurs directly in care work. But the spending also increases employment indirectly in industries that supply the care sector with goods and services and in consumer goods industries as these workers spend their incomes on consumption. Thus, job creation per dollar spent is higher in care than in construction, making it a more effective stimulus.

An expanded system of early care and education is also *job enabling*. By providing increased access to good quality child care staffed by workers who are paid fairly, more mothers of young children will be able to seek and hold jobs. Currently, 64% of mothers with children under the age of six are in paid employment. Among women with children ages 6 to 17, 74% are employed – with the higher employment rate due in large measure to access to public education for children six and older. If an expanded early childhood education system leads to an increase in the share of employed mothers of young children from 64% to 70%, this would add a million workers to the workforce. Some mothers who work part-time because they cannot arrange care of young children or after-school care for older children could increase their hours to full time. If half did this, it would have the same effect as adding another 1.2 million women to the labor force. An increase of 2.2 million workers would result in an annual increase of about $27.4 billion in real GDP.

Finally, investment in quality education for children 0 to 5 years of age *yields a significant positive rate of return*. This is especially true of high-quality programs that enroll young children from economically disadvantaged families. Research (most notably, the evidence from the Perry Preschool Project) has found that every dollar invested yields $7 to $12 back to society.

In a welcome move, on July 21, presidential candidate Joe Biden released his early childhood education plan. Highlights: it provides care for children 0 to 5 years of age with prekindergarden education for 3- and 4-year-olds, caps parents' fees at 7% of income, increases subsidies for low-income families; raises pay and access to training for workers, builds new facilities, and creates 1.5 million new early childhood education jobs.

Eileen Appelbaum, Center for Economic and Policy Research, Washington, DC, USA.

